# Clinical and Echocardiographic Profile of Patients With Heart Failure With Reduced Ejection Fraction: A Retrospective Outcome Analysis

**DOI:** 10.7759/cureus.95354

**Published:** 2025-10-24

**Authors:** Shahul Irfan, Vikhnesh Padmakaran, Ram Prasanth, Sharefa Nazar, Sachith Srinivas, Akshay K TV, Vaibhav S Bhandary, Parth Vinod Patel, Rutuja Baldota, Archana Puthenveedu Soman, Bhagyalakshmi A Vattapothy

**Affiliations:** 1 Internal Medicine, Periyar Government Hospital (Affiliated to Madras Medical College), Chennai, IND; 2 Internal Medicine, Barnsley Hospital NHS Foundation Trust, Barnsley, GBR; 3 Cardiothoracic Vascular Surgery, Dr. KM Cherian Institute of Medical Sciences, Kallissery, IND; 4 Internal Medicine, Subbaiah Institute of Medical Sciences, Shivamogga, IND; 5 Internal Medicine, Narayana Institute of Cardiac Sciences, Bengaluru, IND; 6 Medicine, Bhooma Hospital, Palanpur, IND; 7 Medicine, Deenanath Mangeshkar Hospital and Research Center, Pune, IND; 8 General Medicine, Azeezia Institute of Medical Sciences and Research, Kollam, IND; 9 Oncology, Baby Memorial Hospital, Calicut, IND

**Keywords:** hfref: heart failure with reduced ejection fraction, lv ejection fraction (lvef), pulmonary hypertension, retrospective observational study, systolic heart failure

## Abstract

Introduction: Heart failure with reduced ejection fraction (HFrEF) is a major contributor to cardiovascular morbidity and mortality worldwide. This condition is associated with delayed diagnosis and high comorbidity burden since patients often present at a younger age with advanced disease. Echocardiography plays a central role in identifying structural abnormalities and prognostic markers. This study aimed to evaluate the clinical and echocardiographic profile of patients with HFrEF and assess outcome predictors in a tertiary care center.

Study: A retrospective observational study was conducted at Madras Medical College from May 2024 to May 2025, including 75 patients diagnosed with HFrEF (left ventricular ejection fraction (LVEF) ≤40%). Clinical data on demographics, comorbidities, and New York Heart Association (NYHA) functional class were collected along with echocardiographic parameters such as left ventricular dimensions, diastolic dysfunction, valvular lesions, right ventricular function, and pulmonary artery systolic pressure (PASP). Outcomes assessed included mortality, hospital stay, and readmission.

Results: The mean age was 58.4 ± 11.2 years, with males comprising 45 patients (60%). Hypertension (42 patients, 56%), diabetes (36 patients, 48%), and ischemic cardiomyopathy (39 patients, 52%) were predominant. Most patients presented in NYHA class III or IV (55 patients, 73%). Echocardiography revealed a mean LVEF of 31.6 ± 5.8, LV dilatation in 48 patients (64%), diastolic dysfunction in 60 patients (80%), and moderate-to-severe mitral regurgitation in 25 patients (33%). Patients with EF <25% had significantly higher mortality (five patients, 15%) and readmission (10 patients, 29%) rates. PASP >40 mmHg independently predicted readmissions, while right ventricular dysfunction and mitral regurgitation were associated with adverse outcomes on univariate analysis.

Conclusions: This study highlights that severe left ventricular dysfunction and elevated PASP are strong prognostic markers in HFrEF. Comprehensive echocardiographic evaluation is essential for risk stratification, and early intervention is crucial to improve outcomes in Indian patients with HFrEF.

## Introduction

Heart failure is a clinical syndrome in which the heart is unable to pump blood efficiently to meet the metabolic demands of the body or to fill adequately during diastole, leading to congestion, poor perfusion, and a cascade of compensatory mechanisms [1]. The global burden of heart failure is mounting: recent estimates suggest that more than 60 million people worldwide are affected, and this number is likely to grow with aging populations and improved survival from acute cardiac events [1]. In low- and middle-income countries, the challenges are even greater, in part due to late presentation, resource constraints, and gaps in healthcare infrastructure [2]. In settings such as India, data from tertiary care centers are essential to better understand the real-world spectrum of heart failure.

Among the various types of heart failure, the subtype characterized by reduced left ventricular ejection fraction (LVEF) is of particular clinical relevance because it involves distinct structural and functional derangements of the left ventricle and is the focus of many guideline-directed therapies. Echocardiography is central to characterizing this subtype, as it provides detailed information on left ventricular dimensions, remodeling patterns, valve lesions, right ventricular function, pulmonary pressures, and diastolic indices [3]. Moreover, there is increasing evidence that baseline echocardiographic parameters, including right ventricular function, may help predict outcomes in patients with reduced ejection fraction [4].

In light of these considerations, we conducted a retrospective observational analysis of patients with heart failure with reduced ejection fraction (HFrEF) who were treated at Madras Medical College between May 2024 and May 2025. The purpose of this study was to delineate the clinical characteristics and detailed echocardiographic findings of this patient group and to examine associations between baseline features and key outcomes such as in-hospital mortality and readmissions. We hypothesized that pulmonary hypertension and right ventricular dysfunction would independently predict adverse outcomes in HFrEF by correlating clinical and imaging data with outcomes. We aim to identify prognostic patterns applicable to our local patient population.

## Materials and methods

This retrospective observational study was conducted in the Department of Cardiology, Madras Medical College, Chennai, India, over a one-year period from May 2024 to May 2025. Institutional Human Ethics Committee of Madras Medical College issued approval NEW/EC/MMC/2321. The study population included patients who were admitted with a clinical diagnosis of heart failure and were subsequently found to have reduced LVEF on echocardiography. All patients above the age of 18 years with a LVEF of 40% or less on transthoracic echocardiography were included. Patients with preserved ejection fraction, congenital heart disease, significant pericardial disease, or incomplete medical records were excluded. The diagnosis of heart failure was made based on clinical presentation supported by imaging findings, in accordance with established guidelines [5].

Data were collected retrospectively from hospital medical records, discharge summaries, and echocardiographic reports. Clinical variables included demographic details such as age and sex, cardiovascular risk factors, past history of myocardial infarction, comorbidities including diabetes and hypertension, presenting symptoms, and functional class at admission. Echocardiographic parameters recorded were LVEF, left ventricular end-diastolic and end-systolic dimensions, wall motion abnormalities, left atrial size, right ventricular function, valvular abnormalities, diastolic dysfunction grade, and estimated pulmonary artery systolic pressure (PASP). All echocardiograms had been performed by experienced cardiologists using standard transthoracic techniques, and measurements were obtained following the recommendations of the American Society of Echocardiography [5]. Left atrial enlargement was defined as a left atrial anteroposterior diameter >40 mm in men and >38 mm in women, measured in the parasternal long-axis view. Left ventricular dilatation was considered present when the left ventricular end-diastolic dimension exceeded 58 mm in men and 52 mm in women, based on sex-specific reference ranges. LVEF was calculated using the modified biplane Simpson’s method. Right ventricular systolic dysfunction was identified when tricuspid annular plane systolic excursion (TAPSE) was <17 mm. PASP was estimated from tricuspid regurgitant jet velocity using the modified Bernoulli equation, with values >40 mmHg considered abnormal. Diastolic dysfunction was graded according to mitral inflow velocities (E/A ratio), tissue Doppler imaging (e′), E/e′ ratio, left atrial volume index, and tricuspid regurgitation velocity, following guideline criteria [5]. Inter-observer variability was assessed and sorted out after discussing with a senior cardiologist. Patients with missing data were excluded from the study.

Outcome measures assessed included duration of hospital stay, requirement for intensive care admission, in-hospital mortality, need for advanced support, such as mechanical ventilation or inotropes, and readmission within the study period. Where available, outpatient follow-up data were also reviewed to assess rehospitalization, clinical progression, and mortality. A total sample size of 75 patients was included, which was considered adequate based on the estimated prevalence of reduced ejection fraction among heart failure patients in prior studies, with a 95% confidence level and 5% margin of error. Statistical analysis was performed using descriptive and inferential methods. Continuous variables were expressed as mean with standard deviation, while categorical variables were presented as percentages. Comparison of outcomes between subgroups, such as ischemic versus non-ischemic etiology, was carried out using the chi-square test for categorical data and Student’s t-test or the Mann-Whitney U test for continuous variables. A p-value of less than 0.05 was considered statistically significant.

## Results

A total of 75 patients with HFrEF were included in the study. The mean age of the cohort was 58.4 ± 11.2 years, with the majority belonging to the 50- to 70-year age group. There were 45 males (60%) and 30 females (40%) in the study population. Hypertension was present in 42 patients (56%), making it the most frequent comorbidity, followed by diabetes mellitus in 36 patients (48%) and dyslipidemia in 21 patients (28%). A positive history of smoking was noted in 24 patients (32%), while 15 patients (20%) reported alcohol use. A history of previous myocardial infarction was documented in 30 patients (40%). With respect to etiology, 39 patients (52%) were diagnosed with ischemic cardiomyopathy, whereas 36 patients (48%) had non-ischemic causes, including dilated cardiomyopathy, hypertensive heart disease, and valvular heart disease. At the time of presentation, the majority were in New York Heart Association (NYHA) functional class III, comprising 34 patients (45%), while 21 patients (28%) were in class IV. Smaller proportions were in class II with 15 patients (20%) and class I with five patients (7%) (Table 1).

**Table 1:** Baseline characteristics of the study population. The values are expressed as numbers and their corresponding percentages. NYHA: New York Heart Association

Characteristic	Number of Patients (Percentage)
Mean Age (years)	58.4 ± 11.2
Males	45 (60%)
Females	30 (40%)
Hypertension	42 (56%)
Diabetes Mellitus	36 (48%)
Dyslipidemia	21 (28%)
Smoking	24 (32%)
Alcohol Use	15 (20%)
Previous Myocardial Infarction	30 (40%)
Ischemic Cardiomyopathy	39 (52%)
Non-ischemic Cardiomyopathy	36 (48%)
NYHA Class I	5 (7%)
NYHA Class II	15 (20%)
NYHA Class III	34 (45%)
NYHA Class IV	21 (28%)

The mean LVEF in the cohort was 31.6 ± 5.8%. Left ventricular dilatation was observed in 48 patients (64%), with a mean left ventricular end-diastolic dimension of 61.2 ± 7.4 mm. Left atrial enlargement was documented in 30 patients (40%). Regional wall motion abnormalities were present in 37 patients (49%), most of whom had ischemic cardiomyopathy. Diastolic dysfunction was identified in 60 patients (80%), with grade I in 15 patients (20%), grade II in 28 patients (37%), and grade III in 17 patients (23%). Moderate-to-severe mitral regurgitation was detected in 25 patients (33%), while tricuspid regurgitation was seen in 18 patients (24%). Right ventricular dysfunction was present in 14 patients (19%), and elevated PASP (>40 mmHg) was documented in 20 patients (27%) (Table 2).

**Table 2:** Echocardiographic characteristics of the study population. LVEF: left ventricular ejection fraction; LA: left atrium; RWMA: regional wall motion abnormality; PASP: pulmonary artery systolic pressure

Characteristic	Number of Patients (Percentage)
Mean LVEF (%) Mean ± SD	31.6 ± 5.8%
LV Dilatation	48 (64%)
LA Enlargement	30 (40%)
RWMA Present	37 (49%)
Diastolic Dysfunction (any grade)	60 (80%)
– Grade I	15 (20%)
– Grade II	28 (37%)
– Grade III	17 (23%)
Mitral Regurgitation (Moderate/Severe)	25 (33%)
Tricuspid Regurgitation	18 (24%)
Right Ventricular Dysfunction	14 (19%)
PASP > 40 mmHg	20 (27%)

Subgroup analysis showed that patients with more severe echocardiographic abnormalities had significantly poorer outcomes in terms of mortality and readmission. Patients with an ejection fraction <25% had a significantly higher in-hospital mortality (15%) and readmission rate (29%) compared to those with an ejection fraction of 25-40% (3% and 7% respectively). Right ventricular dysfunction was associated with higher readmission rates, though the difference in mortality did not reach statistical significance. Pulmonary hypertension (PASP >40 mmHg) was strongly associated with readmission, while moderate-to-severe mitral regurgitation correlated significantly with readmission risk (Table 3).

**Table 3:** Correlation between echocardiographic parameters and outcomes. EF: ejection fraction; PASP: pulmonary artery systolic pressure; χ²: chi-square value; *: p < 0.05

Parameter & Subgroup	Mortality n (%)	χ² (Mortality)	p-value (Mortality)	Readmission n (%)	χ² (Readmission)	p-value (Readmission)
Left Ventricular Ejection Fraction - EF <25% (n=34)	5 (15%)	4.21	0.04*	10 (29%)	6.58	0.01*
Left Ventricular Ejection Fraction - EF 25–40% (n=41)	1 (3%)			3 (7%)		
Right Ventricular Function - Dysfunction (n=14)	2 (14%)	1.58	0.21	5 (36%)	5.12	0.02*
Right Ventricular Function - Preserved (n=61)	3 (5%)			7 (11%)		
PASP >40 mmHg (n=20)	3 (15%)	1.79	0.18	7 (35%)	6.72	0.01*
PASP ≤40 mmHg (n=55)	3 (5%)			5 (9%)		
Mitral Regurgitation - Moderate/Severe (n=25)	3 (12%)	0.91	0.34	8 (32%)	5.34	0.02*
Mitral Regurgitation - None/Mild (n=50)	3 (6%)			5 (10%)		

On multivariate logistic regression analysis including LVEF, right ventricular function, PASP, and mitral regurgitation, only ejection fraction <25% and PASP >40 mmHg emerged as independent predictors of adverse outcomes. Ejection fraction <25% was significantly associated with both mortality and readmission, while PASP >40 mmHg independently predicted readmission. Right ventricular dysfunction and moderate-to-severe mitral regurgitation were associated with outcomes on univariate analysis but did not retain statistical significance after adjustment (Table 4).

**Table 4:** Predictors of mortality and readmission (multivariate logistic regression). EF: ejection fraction; RV: right ventricular; PASP: pulmonary artery systolic pressure; MR: mitral regurgitation; OR: odds ratio; CI: confidence interval; *: p<0.05 (statistically significant)

Parameter	Mortality OR (95% CI)	p-value	Readmission OR (95% CI)	p-value
EF <25% vs 25–40%	4.9 (1.2–19.8)	0.03*	3.5 (1.3–9.2)	0.01*
RV Dysfunction	2.1 (0.6–7.9)	0.24	2.8 (0.9–8.3)	0.07
PASP >40 mmHg	3.6 (0.9–13.7)	0.07	4.7 (1.6–13.4)	0.004*
Moderate/Severe MR	1.8 (0.5–6.4)	0.32	2.3 (0.9–6.0)	0.08

## Discussion

HFrEF represents a complex clinical syndrome with heterogeneous etiologies, comorbidity profiles, and prognostic factors. In the present cohort of 75 patients admitted with HFrEF at Madras Medical College, the mean age was 58.4 years, with a predominance of males and ischemic cardiomyopathy as the leading etiology. This is consistent with prior Indian registries such as the Trivandrum Heart Failure Registry, which demonstrated a relatively younger mean age of heart failure onset in India compared to Western cohorts, coupled with a high prevalence of risk factors like hypertension and diabetes [6]. Our findings of hypertension in 42 patients (56%) and diabetes in 36 patients (48%) mirror the study, which reported that comorbid metabolic disease significantly influenced outcomes in South Asian populations [7]. Unlike Western cohorts such as the Framingham Heart Study, where obesity and atrial fibrillation are more prominent comorbidities [8], our population reflects the epidemiologic transition in India, with ischemic cardiomyopathy being the predominant etiology and a high burden of vascular risk factors. The observation that 55 patients (73%) presented with NYHA class III or IV symptoms highlights a tendency for delayed diagnosis and presentation, a pattern described in earlier Indian observational studies as well [9].

Echocardiographic characterization in our study revealed a mean LVEF of 31.6%, with left ventricular dilatation present in 48 patients (64%) and diastolic dysfunction in 60 patients (80%). This is comparable to the European Society of Cardiology Heart Failure Long-Term Registry, where mean EF was approximately 32% and diastolic abnormalities were frequently encountered [10]. The prevalence of moderate-to-severe mitral regurgitation (25 patients, 33%) and tricuspid regurgitation (18 patients, 24%) in our study is in line with prior reports emphasizing the burden of secondary valvular lesions in HFrEF [11]. Interestingly, the prevalence of right ventricular dysfunction (14 patients, 19%) was slightly lower than the 25-35% reported in larger South Indian studies [12]. This difference may be attributable to varying echocardiographic definitions, sample size, and underlying etiologic spectrum. Elevated PASP (>40 mmHg) was found in 20 patients (27%) of our cohort, echoing prior studies that demonstrated pulmonary hypertension as a frequent complication of chronic systolic dysfunction [13]. Collectively, these findings reinforce the role of comprehensive echocardiographic evaluation not only in quantifying EF but also in delineating chamber remodeling, valvular competence, and pulmonary pressures, all of which bear prognostic significance.

Correlation of echocardiographic abnormalities with outcomes revealed that EF <25% was strongly associated with both in-hospital mortality (five patients, 15%) and readmission (10 patients, 29%), compared to significantly lower rates in those with EF 25-40%. This aligns with the Candesartan in Heart Failure Assessment of Reduction in Mortality and morbidity (CHARM) trial, which established that progressively lower EF correlates with higher morbidity and mortality [14]. Our results also underscore the prognostic relevance of pulmonary hypertension: patients with PASP >40 mmHg had a higher readmission rate (35% vs 9%), confirming earlier evidence that pulmonary hypertension is an independent driver of recurrent decompensation in HFrEF [15]. Right ventricular dysfunction was significantly associated with higher readmission but did not predict mortality in our study. Prior work by Ghio et al. has emphasized right ventricular dysfunction as a critical prognostic factor [16], and our divergence may be related to a smaller cohort size and reduced statistical power. Similarly, moderate-to-severe mitral regurgitation was associated with increased complications and readmissions on univariate analysis but lost significance in multivariate regression. This finding is supported by the work of Trichon et al., who demonstrated that mitral regurgitation is often a surrogate marker of advanced left ventricular dilatation and dysfunction rather than an independent predictor when adjusted for other variables [17,18].

Multivariate regression analysis in our study confirmed EF <25% as the strongest independent predictor of mortality and readmission, while PASP >40 mmHg independently predicted readmission. The lack of independent significance for right ventricular dysfunction and mitral regurgitation highlights the overlapping pathophysiology, wherein valvular regurgitation and right heart impairment frequently coexist with severe left ventricular dysfunction and pulmonary hypertension. This is consistent with analyses from the Acute Decompensated Heart Failure National Registry (ADHERE) registry, which showed that after adjusting for EF and hemodynamic indices, the incremental predictive value of mitral regurgitation and right ventricular dysfunction is attenuated [19]. Nevertheless, their presence remains clinically important as markers of advanced disease, warranting close follow-up and potentially earlier intervention. The implications for clinical care are that patients with severely reduced EF and elevated pulmonary pressures represent a high-risk subgroup requiring intensified therapy, guideline-directed medical therapy optimization, and consideration of device-based or advanced interventions.

Our findings also highlight key differences between Indian and Western heart failure populations. The younger age at presentation and higher prevalence of ischemic cardiomyopathy in our cohort is a recurring theme in Indian studies [6,7] and contrasts with Western registries such as CHARM, where non-ischemic etiologies and older age groups are more prominent [14]. These differences underscore the need for region-specific data to guide management strategies, particularly in resource-limited settings. Moreover, the significant readmission burden associated with pulmonary hypertension in our study suggests that closer attention to right heart and pulmonary hemodynamics is warranted in Indian patients, where delayed diagnosis often allows secondary complications to evolve.

The strengths of our study include the systematic echocardiographic evaluation using guideline-based definitions and the integration of both univariate and multivariate statistical approaches to identify prognostic predictors. 

Limitations

This includes the retrospective design, single-center nature, and relatively small sample size, which may reduce statistical power and limit generalizability. Follow-up was limited to in-hospital and short-term outcomes; long-term prognostic implications remain to be studied. The absence of multivariate control for treatment differences, the absence of longitudinal outcome validation, and selection bias (in-hospital patients only) are also the limitations of this study. Despite these limitations, our study contributes valuable real-world evidence regarding the clinical and echocardiographic spectrum of HFrEF in an Indian tertiary care setting.

## Conclusions

This retrospective study from Madras Medical College provides important insights into the clinical and echocardiographic profile of patients with HFrEF in an Indian tertiary care setting. The cohort was relatively young, with a predominance of males and ischemic cardiomyopathy as the leading etiology, and most patients presented with advanced NYHA functional class, reflecting delayed diagnosis and a high comorbidity burden. Echocardiographic evaluation revealed widespread structural abnormalities, with left ventricular dilatation, diastolic dysfunction, and secondary valvular lesions being highly prevalent. Right ventricular dysfunction and pulmonary hypertension were also common, highlighting the multi-chamber involvement typical of advanced HFrEF. Outcome analysis demonstrated that patients with ejection fraction <25% and PASP >40 mmHg had significantly higher mortality and readmission rates, underscoring their prognostic importance. Although mitral regurgitation and right ventricular dysfunction were associated with adverse outcomes on univariate analysis, they did not retain statistical significance after multivariate adjustment, likely reflecting overlap with severe left ventricular dysfunction and elevated pulmonary pressures. Overall, this study emphasizes the value of comprehensive echocardiographic assessment in risk stratification and management of HFrEF, and highlights the need for earlier detection and aggressive optimization of therapy to improve outcomes in the Indian population.
